# Idiopathic ovarian vein thrombosis in a nonpregnant premenopausal woman in her 40s: A case report

**DOI:** 10.1016/j.crwh.2025.e00769

**Published:** 2025-11-27

**Authors:** Ibrahim Hersi, Lubabah Al-Ani, Mehtab Shah, Mohamed Elmezaien, Rehmani Jawad, Olumide Ofinran

**Affiliations:** The Royal Wolverhampton NHS Trust, Wolverhampton WV10 0QP, UK

**Keywords:** Ovarian vein thrombosis, Pelvic pain, Diagnostic imaging, Anticoagulation

## Abstract

Ovarian vein thrombosis (OVT) is a rare and often underdiagnosed condition, most frequently associated with pregnancy, infection, malignancy, or recent surgery. Idiopathic OVT (i.e. that occurring in the absence of identifiable risk factors) is exceedingly uncommon and can easily be overlooked due to its non-specific presentation. This article reports the case of a 40-year-old multiparous woman who presented with a 6-day history of severe left-sided lower abdominal pain associated with fever and nausea. Clinical examination demonstrated left iliac fossa tenderness without palpable adnexal masses. Contrast-enhanced computed tomography (CT) showed a bulky left ovary and left ovarian vein, initially reported as suggestive of left adnexal torsion. Given the presumed diagnosis, diagnostic laparoscopy was undertaken and revealed a thrombosed left ovarian ligament without torsion, leading to left partial salpingectomy. Postoperatively, the final CT report concluded that the appearances were highly suggestive of left ovarian vein thrombosis. The patient was commenced on anticoagulation following haematology consultation. This case demonstrates that OVT may arise in the absence of known risk factors and highlights the diagnostic challenges when it mimics more common acute pelvic pathologies. Careful radiological review, multidisciplinary involvement, and a high index of suspicion are essential to avoid unnecessary surgical intervention and ensure timely anticoagulation in stable patients.

## Highlights

• Idiopathic ovarian vein thrombosis is an important but exceedingly rare cause of acute pelvic pain in women.
• Ovarian vein thrombosis can mimic adnexal torsion and prompt unnecessary surgery.
• Timely imaging facilitates accurate diagnosis and may prevent laparoscopy.
• Multidisciplinary input is key to achieving optimal medical management.

## 1 Introduction

Ovarian vein thrombosis (OVT) is a rare thromboembolic condition that primarily affects postpartum women or those with other hypercoagulable states such as malignancy or infection [1]. Idiopathic OVT (i.e. that occurring in the absence of identifiable risk factors) is exceedingly uncommon. OVT typically presents with non-specific symptoms, including abdominal pain and fever, which overlap with several acute abdominal pathologies, making diagnosis challenging [2]. In patients without risk factors, clinical suspicion may be even lower, increasing the likelihood of misdiagnosis. If not promptly recognised and treated, fatal complications such as pulmonary embolism may occur [2].

This report concerns a case of left-sided idiopathic OVT initially misdiagnosed as ovarian torsion, resulting in surgical intervention. It highlights the diagnostic challenges associated with idiopathic OVT and the importance of multidisciplinary input to ensure timely anticoagulation and avoid unnecessary surgery.

## 2 Case Presentation

A 40-year-old multiparous woman presented to the emergency department with a 6-day history of severe left-sided lower abdominal pain, nausea and fever. She had a history of two caesarean sections and one vaginal delivery. The pain was sharp, sudden in onset, persistent and worsened by sitting, reaching a severity of 9/10. She denied gastrointestinal and genitourinary symptoms. She was on day 21 of a regular 28-day menstrual cycle. She was sexually active with one regular partner without contraception. She was a non-smoker with no recent immobility and no personal or family history of thrombosis.

On examination, vital signs were stable. A urine pregnancy test was performed and was negative. There was localised left iliac fossa tenderness with voluntary guarding but no rebound. Pelvic examination revealed left adnexal tenderness with no palpable masses. There were no signs of deep vein thrombosis in the extremities. Laboratory tests were unremarkable apart from an elevated C-reactive protein (236 mg/L).

**Fig. 1:**
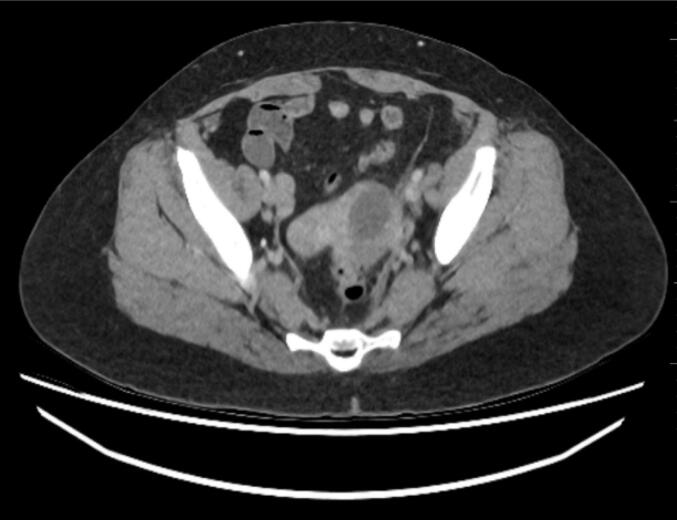
Axial contrast-enhanced CT demonstrating thick-walled cyst in the left ovary.

**Fig. 2:**
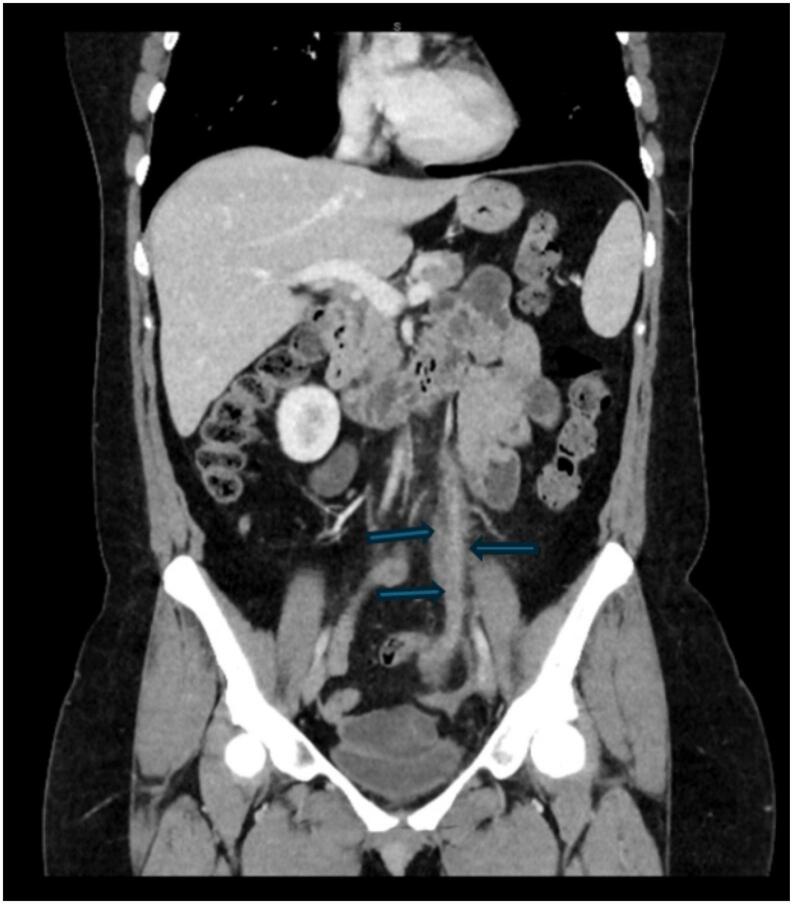
Coronal reconstructed contrast-enhanced CT image demonstrating distended poorly enhancing left ovarian vein (arrow) with subtle surrounding inflammatory reaction. The distal most segment of the left ovarian vein is normal calibre and enhances.

**Fig. 3:**
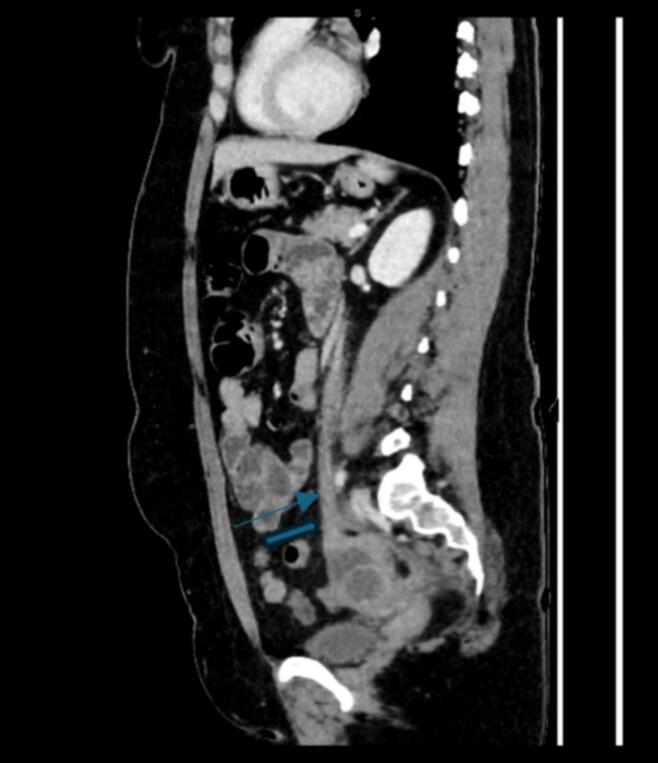
: Sagittal reconstructed contrast-enhanced CT image demonstrating poorly enhancing left ovarian vein (thin arrow), and partially seen left ovarian cyst (thick arrow).

**Fig. 4:**
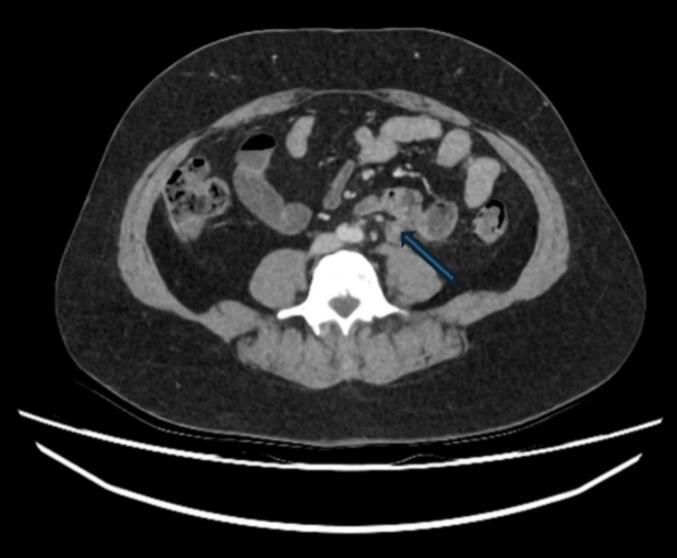
Axial contrast-enhanced CT image demonstrating distended poorly enhancing left ovarian vein (arrow).

Contrast-enhanced computed tomography (CT) was performed. A transvaginal ultrasound scan was not undertaken as CT was readily available and expedited due to the diagnostic concern for adnexal torsion. As this was an out-of-hours case, a provisional report was provided by a resident radiology physician, which concluded that there were features highly suspicious for left ovarian torsion (Fig. 1, Fig. 2, Fig. 3, Fig. 4). Diagnostic laparoscopy revealed a thrombosed left ovarian ligament without torsion, as well as an oedematous and inflamed lateral left fallopian tube, prompting a left partial salpingectomy.

Postoperatively, the final CT report concluded that the findings were more consistent with left haemorrhagic ovarian cysts, with a high suspicion for left OVT. The patient received intravenous antibiotics for three days and was commenced on apixaban following haematology review. Thrombophilia testing was negative, and histopathology showed acute inflammation.

When reviewed in the haematology clinic 7 months later, the patient reported discontinuing apixaban after 3 months due to pill fatigue but had remained well in the elapsed time. Planned gynaecology follow-up to review symptoms and consider repeat imaging did not occur, as she did not attend appointments, and contraception counselling could not be provided.

## 3 Discussion

This case raises important considerations regarding the presentation, diagnosis and management of OVT. This rare condition is characterised by the presence of a thrombus within the ovarian vein. Its incidence is estimated to be 60-fold lower than lower limb deep vein thrombosis [3]. Previous studies have reported risk factors for OVT which are classically associated with hypercoagulability, including pregnancy, infections, malignancy, the use of oral contraceptives and recent surgical procedures.

OVT is most associated with the postpartum period, with an incidence of 0.18 % and 2 % with vaginal births and caesarean sections, respectively [2]. This association can be attributed to pregnancy-related changes encompassing Virchow's triad: venous stasis from the gravid uterus, increased circulating oestrogen contributing to hypercoagulability, and endothelial injury from the pro-inflammatory state of pregnancy and labour [4]. However, in this case, the absence of any apparent risk factors led to the diagnosis of idiopathic OVT, which is estimated to account for less than one-fifth of all cases [5].

OVT is often overlooked as a cause of acute abdominal pain because its presentation overlaps with more common conditions such as appendicitis, pelvic inflammatory disease or ovarian torsion. The typical presentation of OVT involves pelvic pain, fever and other nonspecific symptoms, including nausea and vomiting [2]. A palpable, cord-like abdominal mass, corresponding to the thrombosed ovarian vein, is considered a classic examination finding, although it is reported in only 46 % of cases. Typically, this mass is right sided, as 70–80 % of cases involve the right ovarian vein, likely due to its longer anatomical course [4]. The patient's clinical presentation was unusual, as she had left-sided abdominal pain and no palpable mass. Given the heterogeneity of initial presentations, a high index of clinical suspicion is essential when diagnosing OVT.

Imaging has a central role in diagnosis. Radiological findings of OVT typically include a filling defect within the ovarian vein on contrast-enhanced CT. If large enough, the thrombus may extend into draining veins (i.e. the left renal vein from a left ovarian vein thrombus). The affected ovarian vein may be enlarged or dilated, with surrounding inflammatory changes such as fat stranding and perivascular oedema. In some cases, there may be evidence of pulmonary embolism as a complication of OVT [5,6].

In this case, CT findings were initially interpreted as suspicious for ovarian torsion, prompting diagnostic laparoscopy. OVT and ovarian torsion share overlapping imaging features, including ovarian enlargement. However, ovarian torsion is more likely to demonstrate displacement of ovarian follicles resulting in a “string of pearls” appearance. A “whirlpool sign”, representing a twisted ovarian pedicle, is pathognomonic for ovarian torsion, seen in approximately 91 % of cases [7,8]. Surrounding inflammatory changes or free fluid in the pelvis is commonly seen, with possible haemorrhage within the adnexal mass. In ovarian infarction, the ovary may show a lack of enhancement on contrast-enhanced CT [9,10].

Contrast-enhanced CT is commonly used as a first-line modality given its availability, speed, and high specificity and sensitivity to detect a thrombus [5,6]. However, a scoping review published in 2024 examining the diagnosis and management of OVT found that magnetic resonance imaging (MRI) has a higher sensitivity (92–100 %) and specificity (100 %) for diagnosing OVT [5,6]. Typical thrombus findings on MRI involve a hyperintense filling defect with loss of normal flow void on T2-weighted images [5,6]. Asymmetric enlargement and oedema of the affected ovary is also commonly seen on MRI [5,11].

Ultrasound is an inexpensive and safe imaging modality. If OVT is present, the ovarian vein appears enlarged, with an intraluminal echogenic thrombus, and colour doppler showing absent, marginal, or decreased blood flow within the affected vein. The downside of ultrasound is its reduced sensitivity for OVT diagnosis and operator-dependent findings, with some studies revealing a 50–56 % detection rate [5,12]. A structured imaging review protocol or MRI could have potentially avoided surgery in this clinically stable patient.

There are no established guidelines for OVT management [13]. Providers are therefore guided by specialist input and often extrapolate from general guidance used in the management of deep vein thrombosis. Treatment involves anticoagulation therapy and antibiotics whilst targeting the underlying cause (e.g. thrombophilia, malignancy, infection, etc.) [6,14]. Historically, warfarin and heparin therapies were used, but direct oral anticoagulants are now commonly utilised [13]. Treatment duration varies, with some case studies reporting up to one year of treatment with warfarin for postpartum OVT, and two to six months of anticoagulation for idiopathic OVT [6,14]. Follow-up imaging to ensure thrombus resolution has been reported in several case studies, with some patients discontinuing anticoagulation therapy after reassuring imaging findings [6,13,14]. The patient in the present case discontinued apixaban after three months of treatment without recurrence of symptoms, though the safety of shorter treatment durations remains uncertain.

## 4 Conclusion

This case highlights the diagnostic and therapeutic challenges of idiopathic OVT, a rare condition that can mimic ovarian torsion and lead to unnecessary surgery. A high index of clinical suspicion, careful imaging review, and multidisciplinary input are essential for timely diagnosis and optimal medical management.

## Contributors

Ibrahim Hersi contributed to patient care, conception of the case report, acquiring and interpreting the data, undertaking the literature review and drafting the manuscript, and revising the article critically for important intellectual content.

Lubabah Al-Ani contributed to acquiring and interpreting the data, undertaking the literature review and drafting the manuscript, and revising the article critically for important intellectual content.

Mehtab Shah contributed to patient care, acquiring and interpreting the data and undertaking the literature review, and revising the article critically for important intellectual content.

Mohamed Elmezaien contributed to patient care, acquiring and interpreting the data, and revising the article critically for important intellectual content.

Rehmani Jawad contributed to acquiring and interpreting the data and revising the article critically for important intellectual content.

Olumide Ofinran contributed to patient care, conception of the case report, acquiring and interpreting the data, and revising the article critically for important intellectual content.

All authors approved the final submitted manuscript.

## Patient consent

Written informed consent was obtained from the patient for publication of the case report and accompanying images.

## Provenance and peer review

This article was not commissioned and was peer reviewed.

## Funding

The authors received no financial support for the publication of this article.

## Declaration of competing interest

The authors declare that they have no competing interest regarding the publication of this case report.
